# Validation by Molecular Dynamics of the Major Components of Sugarcane Vinasse, On a Surface of Calcium Carbonate (Calcite)

**DOI:** 10.3390/molecules26082353

**Published:** 2021-04-18

**Authors:** Oscar Eduardo Rojas Álvarez, María Inés Nicolás Vázquez, Jose Oñate-Garzón, Carlos A. Arango

**Affiliations:** 1 Grupo de Investigación en Química y Biotecnología (QUIBIO), Universidad Santiago de Cali, Calle 5 # 62-00, Cali 760004, Colombia; jose.onate00@usc.edu.co; 2 Doctorado en Ciencia Naturales para el Desarrollo (DOCINADE), Instituto Tecnológico de Costa Rica, Universidad Nacional—Universidad Estatal a Distancia, San José 40101, Costa Rica; nicovain@yahoo.com.mx; 3 Departamento de Ciencias Químicas, Facultad de Estudios Superiores Cuautitlán, Campo 1, Universidad Nacional Autónoma de México, Cuautitlán Izcalli, Estado de Mexico C.P. 54740, Mexico; 4 Departamento de Ciencias Químicas, Facultad de Ciencias Naturales, Universidad Icesi, Calle 18 # 122-135, Cali 760031, Colombia; caarango@icesi.edu.co

**Keywords:** vinasse, molecular dynamics, calcite, calcium carbonate, mannoprotein

## Abstract

There is ongoing interest in the alcohol industry to significantly reduce and/or add value to the liquid residue, vinasse, produced after the distillation and rectification of ethanol from sugar cane. Vinasse contains potassium, glycerol, and a protein component that can cause environmental issues if improperly disposed of. Currently, some industries have optimized their processes to reduce waste, and a significant proportion of vinasse is being considered for use as an additive in other industrial processes. In the manufacture of cement and asphalt, vinasse has been used in the mixtures at low concentrations, albeit with some physical and mechanical problems. This work is the first molecular approximation of the components of the sugar cane vinasse in an industrial context, and it provides atomic details of complex molecular events. In the current study, the major components of sugar cane vinasse, alone or complexed on the surface of calcium carbonate, were modeled and simulated using molecular dynamics. The results showed that the protein component, represented by the mannoprotein Mp1p, has a high affinity for forming hydrogen bonds with potassium and glycerol in the vinasse. Additionally, it provides atomic stability to the calcium carbonate surface, preserving the calcite crystalline structure in the same way potassium ions interact with the carbonate group through ion–dipole interactions to improve the cohesion of the modeled surface. On the contrary, when the glycerol molecule interacts with calcium carbonate using more than two hydrogen bonds, it triggers the breakdown of the crystalline structure of calcite expanding the ionic pair.

## 1. Introduction

Ethanol production from sugarcane is a thriving industry in tropical areas. The alcohol industry is expanding in proportion to population growth as consumption of chemical products, using ethanol as a diluent, follows population growth. However, as ethanol production increases, so does its by-product, vinasse, which is generated at a rate of 13 L for every liter of ethanol produced regardless of the industry [1,2,3]. The inadequate and indiscriminate disposal of sugarcane vinasse brings with it environmental problems [4].

Vinasse is the liquid residue left after the distillation and rectification of ethanol. It is dark brown in color, with a high content of organic matter, acidic in nature, and has a characteristic odor [5]. Physicochemical and microbiological characterizations conducted on vinasse obtained from distilleries in Brazil, Cuba, and Colombia show that the main components of sugarcane vinasse are as follows: the potassium ion (K^+^) as the major mineral component, glycerol as an intermediate product of alcoholic fermentation, and glutamic acid as the major amino acid [5,6] of cell wall proteins in yeast.

Currently, the alcohol industry has adopted several alternatives to modify the ethanol production process to make it more efficient. Among these alternatives, the use of reboilers in the distillation process allows a reduction of approximately 16% in vinasse production [7]. Despite these efforts, population growth and expanding automobile use continue to lead to increased production of vinasse, and the environmental impact of its storage or its traditional uses, such as fertigation, continue to cause environmental issues in water and soil quality [1].

The problems associated with vinasse production have led to the industry considering alternative uses for the by-product. A promising avenue for the utilization of vinasse is its potential use as an additive in cement and asphalt production [8,9]. Cement is mainly formed by a mixture of limestone and clay in a conglomerating form, where calcite predominates as a mineral formed by calcium carbonate (CaCO_3_).

It has been reported that where cement mixtures were made with stillage concentrations lower than 10%, the physicochemical characteristics of the product were acceptable [8,9], but at higher percentages or varying the concentrations of stillage components, the physicochemical attributes of either asphalt or cement may be adversely affected. This is due, in part, to the fact that the molecular behavior of the asphalt/cement components when interacting with vinasse is unknown.

This study aimed to model, validate, and molecularly simulate stable chemical complexes formed among the components of vinasse, glutamic acid, glycerol, mannoprotein Mp1p, and surfaces containing CaCO_3_ (the main compound used in cement manufacture). This study is also with the purpose of providing molecular knowledge, for the creation of more efficient and responsible additives with the marketed product and the environment.

## 2. Models and Computational Methods

### 2.1. Three-Dimensional Structures

The molecular modeling of each chemical complex (Table 1) was conducted to obtain their three-dimensional (3D) structures. Each 3D structure was packed in a data file with the extension PDB as follows: 1. Glycerol was obtained from the ChEMBL database (ID: CHEMBL692) [10] and was structurally related to the molecule with the code GOL, which was obtained from the Protein Data Bank in Europe (PDBe) database [11]. 2. Glutamic acid was obtained from the ChEMBL database (ID: CHEMBL575060) [10] and was structurally related to the molecule with the code GLU, which was obtained from the PDBe database [11]. 3. Molecule of the antigenic cell wall mannoprotein (Mp1p) was obtained from the Research Collaboratory for Structural Bioinformatics—Protein Data Bank (RCSB PDB) database [12] (ID: 3L1N) and was complexed with palmitic acid. 4. CaCO_3_ ionic pair was obtained from the DrugBank database [13], under the accession number DB06724. 5. Crystalline structure of calcite was downloaded from ChemTube3D, provided by the University of Liverpool by Professor Nick Greeves.

### 2.2. Molecular Modeling

The Mp1p protein structure contained in the 3L1N data file was isolated from palmitic acid and molecularly modeled by changing selenomethionine (MSE) to methionine (MET); this was achieved by changing selenium into the S3 type sulfur atom, SD. The CG-SD and SD-CE bond lengths were set to 1.81 and 1.78 Å, respectively. This conversion of residues is important because the load charges and other parameters were available for MET, but not for MSE [15]. Furthermore, the protein in its natural state most likely contains MET instead of MSE since proteins are generated with MSE because Se atoms are useful in determining the X-ray structure [20].

#### 2.2.1. Molecular Optimization Using DFT

The chemical structure of CaCO_3_ and the crystalline structure of calcite, made up of four calcium ions in electrostatic interaction with six carbonate groups, were analyzed at the quantum mechanical level. The geometry optimization in the ground state was calculated applying the DFT [21,22] using Gaussian09 software [23] (Krone et al., 2012). These calculations were performed using Becke’s three-parameter hybrid functional (B3LYP) [24,25], which includes a mixture of the Hartree–Fock exchange with the DFT exchange correlation. The 6-31G (d) basis set that includes the split-valence and polarization function was used [26]. For the calculation of neutral ionic pair, a charge of zero and spin-singlet was used. The calculation of the Merz–Kollman electrostatic potential (MK-ESP) charges was performed at a single point [27,28].

The surface of CaCO_3_ was modeled using the optimized structure from the DFT calculations. Four hundred ionic pairs of CaCO_3_ were arranged in a box of 20 × 80 × 80 Å with a tolerance of 2.0 using the Packmol software [29].

#### 2.2.2. Molecular Optimization Using CHARMM22

The validation of the molecular structures making up the chemical complexes was performed by a computational comparison of the 3D structures with the force fields listed in Table 1 using Assisted Model Building with Energy Refinement (AMBER) [30]. The chemical complexes containing CaCO_3_ and Calcite on their surfaces were subjected to the Merck Molecular Force Field, and the Van der Waals parameters were obtained from Chemistry at Harvard Macromolecular Mechanics (CHARMM22) [31] (Table 1).

Validation of the glycerol molecule was necessitated by the possibility of significant differences between the conformations. In one conformation, the rotation of one of the carbons in the O1-C1-C2-O2 dihedral puts the oxygens in a trans conformation. The orientation of polar hydrogens is also important for the electrostatic field of glycerol, and in a simulation, they can reasonably be assumed to rotate strongly depending on the orientation of nearby water molecules.

### 2.3. Molecular Orientation

The orientation of the molecules to form the simulated complexes was developed for the GOL-GLU-K complex using the R-side-chain of glutamic acid, allowing for attractive and repulsive interactions. The hydrogens of carbon 1 (C1) in glycerol were oriented with the hydrogens of carbon beta of the R-side-chain of glutamic acid, allowing for a repulsive interaction at a distance of 1.84 Å. Similarly, the hydrogens of carbon 3 (C3) in glycerol were oriented with the unbound oxygen of the last carbon (CD) of the R-side- chain, allowing for an attractive interaction at a distance of 1.89 Å. Molecular orientation was developed through the R-chain of glutamic acid since the carboxyl (COOH) and amino (NH_2_) groups are responsible for the formation of proteins, and these groups would be linked in a peptide [32].

The orientation of the GOL-3L1N-K complex, consisting of Mp1p with the glycerol molecule, was developed by identifying the most active regions on the mannoprotein that bind to small molecules used as probes using the FTMap (computational solvent mapping) webserver, which uses molecules as probes to predict binding hot spots [33]. The most active zone in the 3L1N mannoprotein was located in the center of three alpha-helices, where the highest percentage of hydrogen bonds between the protein and the probe molecules was determined by serine and glutamine. The information obtained from the FTMap allowed for the orientation of the glycerol molecule into polar groups.

### 2.4. Solvation Process

All molecular complexes were solvated using water following the theTIP3P solvation models in a water box TIP3PBOX [34]. Water molecules were added 10 Å away from the last atom of each molecular structure in three dimensions.

The data on parameters and topologies were qualitatively analyzed using the Visual Molecular Dynamics software [35], and the spatial orientations for developing molecular complexes were conducted using Swiss-PdbViewer (aka DeepView) [36].

The simulations for 3A to 4D were configured in NAMD-Scalable Molecular Dynamics, which is a parallel molecular dynamics code designed for high-performance simulations [37] (Table 1). All simulations were solvated in a water box with an implicit solvent of the TIP3P water model, while the molecular dynamics simulation was conducted using Periodic Boundary Conditions and Particle Mesh Ewald.

### 2.5. Simulation Settings

All simulations were configured using energy minimization, and equilibration was done in 100 steps with the distance beyond which electrostatic and Van der Waals interactions were cut-off at 12 Å. Each molecular simulation lasted for 3000 ps at 1 fs per step. Temperature and pressure were held at 310 K and 1 atm, respectively.

The minimization and equilibration simulations generated trajectories files that were analyzed by computing the root-mean-square deviation (RMSD) [38].

## 3. Results and Discussion

### 3.1. Chemical Complexes of the Major Sugarcane Vinasse Components

Previous studies have shown that sugarcane vinasse is generally composed of potassium (K), an ionic element, glycerol (GOL), a major intermediate of alcoholic fermentation, and protein derived from *Saccharomyces cerevisiae* as a polymer complex, where glutamic acid (GLU) predominates as the constitutive amino acid of membrane proteins (3L1N) [6,39].

**Figure 1:**
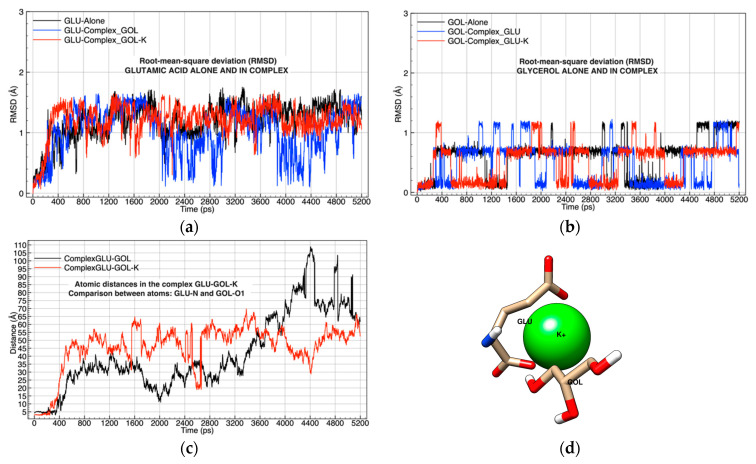
Analysis of the molecular dynamics of the glutamic acid (GLU)–glycerol (GOL)–potassium (K) complex: (**a**) root-mean-square deviation (RMSD) compared to the simulation of GLU without ligands; (**b**) RMSD compared to the simulation of GOL without ligands; (**c**) analysis of atomic distances between nitrogen in GLU and oxygen 1 in GOL in two simulations, one influenced by K and the other without K; (**d**) graphic representation of the GLU-GLO-K complex, oxygen atoms in red, carbons in gold, hydrogen in white, nitrogen atom in blue, and potassium ion in green, image taken at 400 ps of molecular dynamics.

Results of the GOL-GLU-K complex simulation (Simulation 1, Table 1), analyzed using RMSD, indicated that glutamic acid atoms deviated but showed greater stability when they were complexed with glycerol and a K^+^. The K^+^ ion achieved stability that showed a deviation of approximately 1 Å compared to the GLU-GOL complex that fluctuated at approximately 1.5 Å (Figure 1a).

On the contrary, the glycerol molecule exhibited transient stability, and its behavior differed between when it was alone and when it was complexed. This implies that the glycerol molecule is electrostatically influenced by glutamic acid and K^+^, and it has periods of extended stability that range from 500 to 1500 ps and 4300–5200 ps when complexed with GLU-K (Figure 1b).

However, an analysis of the distances between the nitrogen atom in glutamic acid and the oxygen atom at carbon 1 of glycerol in the GLU-GOL and GLU-GOL-K complexes indicated that the distances exceeded the Van der Waals forces, suggesting that there was no interaction among these three chemical species (Figure 1c).

It is possible that the low attraction observed among glutamic acid, K^+^, and glycerol strengthens the likelihood that the amino acids in vinasse form natural polymers [6,40] that allow stillage to maintain physical stability in its liquid and solid forms [8].

**Figure 2:**
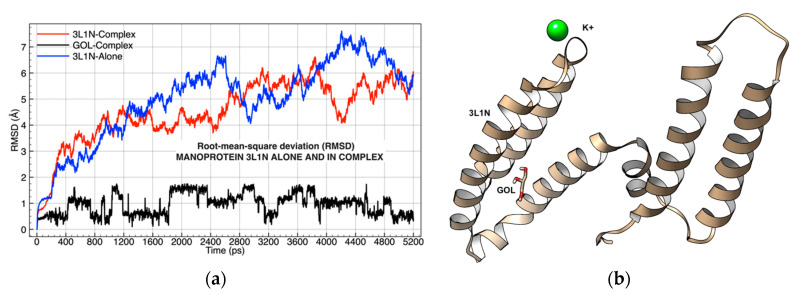
(**a**) Root-mean-square deviation (RMSD) of the mannoprotein (3L1N), simulated in a complex with GOL-K and without ligands; (**b**) graphical representation of the 3L1N-GOL-K complex after energy minimization, starting point of Simulation 2C, oxygen atoms in red, carbons in gold, hydrogen in white, nitrogen atom in blue, and potassium ion in green.

Simulation 2 of the GOL-3L1N-K complex (Table 1), analyzed using RMSD, showed that glycerol and K^+^ had different atomic deviations, which meant that the molecular structure of the protein was influenced by the glycerol ligand (Figure 2a).

Glycerol also behaved differently, and its atomic deviations from its center of mass were close to the mannoprotein, thus remaining under its attractive forces for long periods during the molecular dynamics simulation.

A qualitative exploration of the simulation of the mannoprotein Mp1p (3L1N) complexed with glycerol (GOL) and K^+^ showed a loss of affinity in the active zone identified by FTMap [33]. However, the complex did not lose affinity within the three alpha-helices (Figure 2a,b).

### 3.2. The Major Components of Sugarcane Vinasse on a Calcium Carbonate Surface

**Figure 3:**
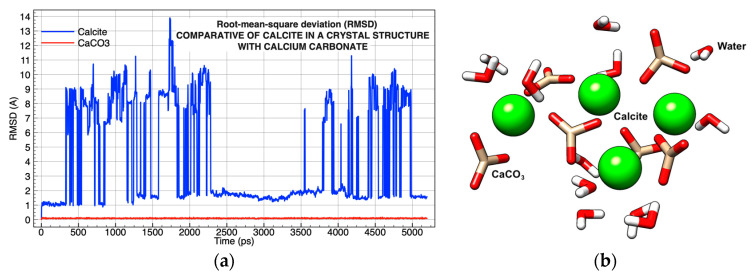
Analysis of the molecular dynamics of the CaCO_3_ ionic pair and the crystalline structure of calcite: (**a**) root-mean-square deviation (RMSD) of the two simulations in comparison; (**b**) image taken of the molecular structure of calcite at 3000 ps simulation, oxygen atoms in red, carbons in gold, hydrogen in white, nitrogen atom in blue, and potassium ion in green.

In Simulation 3, the calcite in a crystalline molecular conformation and the calcium carbonate ionic pair were investigated to determine which structure would be used as a starting point for the conformation of the surface of the CaCO_3_ molecular model (Table 1). The calcite crystals and CaCO_3_ ionic pairs were solvated in two water boxes. The analysis of RMSD after 5000 ps showed a high molecular deviation of the crystalline structure because the solvation process using water competed with the force from the crystal surface, affecting the dielectric constant of water [41] (Figure 3a).

Conversely, ionic pairs of CaCO_3_ showed high stability (Figure 3a) making them the best candidate to conform to the surface. The strong binding between the calcium and the carbonate (∼8 kcal/mol) in water [42] allowed the structure to remain intact, but it did not prevent the oxygen in the water molecules from interacting strongly with calcium. It was this interaction that allowed for the partial solubility of CaCO_3_ and the breakage of the calcite’s crystalline structure (Figure 3b).

The molecular dynamics simulation took place during the first 300 ps, maintaining the molecular structure of the calcite, implying that all the CaCO_3_ ionic pairs made up the crystalline structure. Shortly afterward, one of the CaCO_3_ ionic pairs was attracted through Ca^2+^ to the water molecules, breaking the crystalline structure and creating the molecular deviations observed at intervals of 300–2250 ps and 3250–5000 ps. However, in the time interval from 2250 to 3750 ps, the six ionic pairs of CO_3_ returned to the spatial conformation of calcite, expelling the water molecules from their conformation (Figure 3a).

Taking the results of Simulation 3 as a reference, the calcium carbonate ionic pair was used as the starting point to create the surface for the simulations since ionic pairs were packed into defined regions of space, guaranteeing the formation of crystalline structures and molecules alone. CaCO_3_ freed spaces for solvation so that the short-range repulsive interactions did not interrupt the simulations.

**Figure 4:**
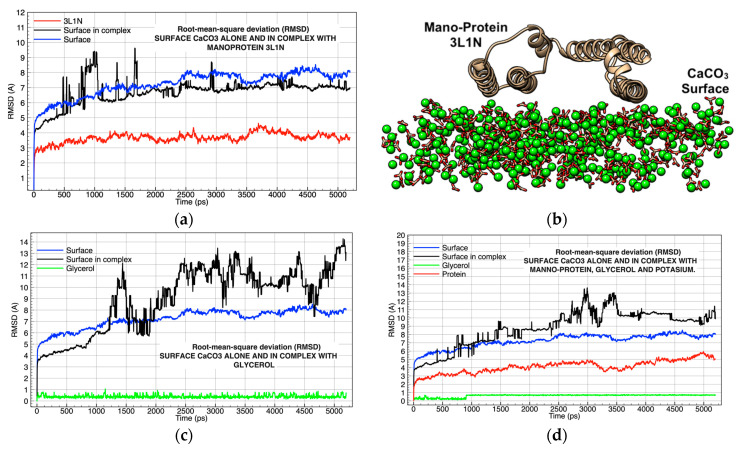
Analysis of the molecular dynamics of the complex surface–glycerol (GOL)–potassium (K): (**a**) root-mean-square deviation (RMSD) compared the mannoprotein (3L1N) on the surface in complex and without ligands; (**b**) graphical representation of the surface-3L1N complex, image taken after orientation of mannoprotein over the surface, oxygen atoms in red, carbons in gold, and potassium ion in green; (**c**) RMSD compared GOL with the surface in complex and without ligands; (**d**) RMSD compared 3L1N-GOL complex with the surface and without ligands.

The last group of simulations (Simulation 4, Table 1) showed that the protein component represented by the mannoprotein 3L1N remained on the surface during the entire simulation, and its molecular interaction was minimal since it did not achieve large deviations on the surface of CaCO_3_ (Simulation 4C; Table 1; Figure 4a,b). Hydrogen bond interactions occurred between the long residues located in the alpha-helix of the mannoprotein, which was in contact with the CaCO_3_ surface, specifically the hydrogens located on the side chain of the residues. Lysine, asparagine, glutamine, and arginine interacted via hydrogen bond interactions with the oxygens in the carbonate group of the surface (C-O----H-N). Additionally, the ion–dipole interactions between the oxygens in the isobutyl group in leucine and Ca^2+^ on the surface (C-O----Ca) were analyzed (Figure 4b).

The opposite case occurred when the glycerol molecule interacted molecularly with the surface of CaCO_3_ during the first 1250 ps of simulation. The glycerol molecule remained inoperative despite the hydrogen bonds on the surface (C-O----H-O) throughout the simulation. The CaCO_3_ ionic pairs making up the surface exhibited an increase in molecular deviation because the glycerol molecules tended to separate the crystalline structure that made up the calcite, reducing particle size in a manner similar to that observed experimentally [43]. This attraction by hydrogen bridges caused the separation and expansion of the surface (Figure 4c).

The final simulation consisting of the glycerol molecule, the mannoprotein 3L1N, and K^+^ on the CaCO_3_ surface (Table 1) indicated that the glycerol, protein, and K^+^ did not show any significant molecular deviations throughout the 5250 ps of molecular dynamics. However, the CaCO_3_ surface endured several intervals of instability that sometimes exceeded 5 Å (Figure 4d).

**Figure 5:**
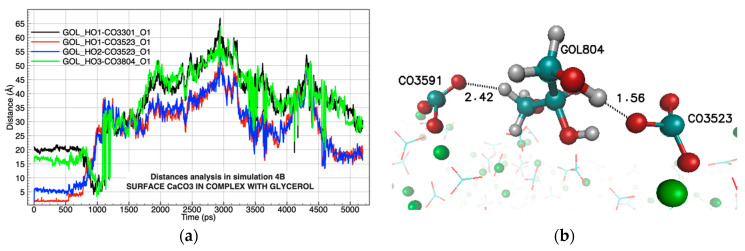
(**a**) Analysis of atomic distances between glycerol (GOL) and the carbonate group (CO_3_) on the surface; (**b**) graphical representation of the distances with the carbonate group that occur from 0 to1000 ps in the simulation, oxygen atoms in red, carbons in blue, hydrogen in white, and potassium ion in green.

A deeper analysis where the distances of the hydrogen bonds were measured in all the molecular dynamics showed that during Simulation 4B (Table 1), the conformation of seven attractive hydrogen interactions occurred at less than 3 Å from the start of the simulation to approximately 1250 ps (Figure 5a). The most representative interaction occurred with an occupation of 8.27% between the glycerol molecule (GOL804) and the carbonate molecule (CO_3_523) (Figure 5b, Table 2).

Finally, an analysis of the hydrogen bonds responsible for the stability of the surface, in a comparison between Simulation 4B and 4D, showed that the protein component (3L1N) remained with eighteen bonds throughout the simulation (Table 2), avoiding the breakdown of the calcite molecular structure and expansion of the CaCO_3_ surface. This molecular behavior is consistent with studies where sugar cane vinasse was used as an additive in cement or concretes with positive results in physicochemical and mechanical properties [8,9,44].

However, the current molecular study showed that glycerol may cause mechanical problems, while K^+^ incorporated into the calcite through ion–dipole interactions together with the mannoprotein allowed for the correct cohesion of the mixture.

It is important to highlight that this research work is constituted as the first molecular approximation of the components of the sugar cane vinasse in an industrial context and provides atomic details of complex molecular events. However, it is an approximation to the reality that has related limitations to each of the techniques used to model, optimize, and dynamize the molecules and complexes used.

## 4. Conclusions

After running twelve simulations, where chemical complexes were developed using the major components found in sugar cane vinasse and their interaction with CaCO_3_ surface, the following conclusions were reached:

The literature currently reports a composition of amino acids in sugar cane vinasse consisting of fragmented yeast proteins as a result of the high temperatures obtained during vinasse production. It is necessary to characterize vinasse proteins, as the results of the current study showed a high affinity of K^+^ and the glycerol molecules to a resistant mannoprotein of the cell wall in yeast.

For the creation of a surface that simulates cement, the current study used the CaCO_3_ ionic pair as a starting point since it had low atomic deviations with respect to the crystalline structure of calcite. It was evident in all the validations of the molecular models and simulations that the calcite crystal is formed and is the most stable structure that prevents the expansion of the surface (cement).

Glycerol produced in the alcohol industry in proportions between 2.5 and 2.7% (*m*/*m*) forms hydrogen bonds with the oxygen components of two CaCO_3_ ionic pairs, forcing it to break the crystalline structure of calcite, triggering surface instability. It is likely that the reported problems of adhesion and drying times in the use of stillage as an additive in asphalt and cement were caused by the glycerol content.

The highest content of vinasse reported corresponds to potassium in ion form, which, depending on the industrial process, is approximately 4% (*m*/*m*). The present study showed that K^+^ is incorporated into calcite through ion–dipole interactions and allows for the correct cohesion of the complex, which makes it a good additive for cement.

Finally, the protein content in vinasse, which falls between 7% and 9%, is essential in the stability of the CaCO_3_ surface and tends to preserve the crystalline structure of calcite. In addition to interacting through hydrogen bonds with the surface, it can form natural polymers that improve the physicochemical properties of cement.

## Figures and Tables

**Table 1:** Description of the simulations carried out, force fields, and solvation processes.

Chemical Complexes Modeled	Simulation	Components	Force Fields	Number of Water Molecules
GOL-GLU-K	1A	GOL	AMBER: General AMBER force field (GAFF) [14] Primary protein model. ff14SB [15]	530
	1B	GLU		605
	1C	GLU-GOL		671
	1D	GOL-GLU-K		670
GOL-3L1N-K	2A	3L1N		12681
	2B	GOL-3L1N		12911
	2C	GOL-3L1N-K		12910
Calcite	3A	Calcite	CHARMM: General FF (CGenFF) [16] Empirical force field parameterization for proteins [17], lipids [18] and carbohydrates [19].	9240
	3B	CaCO_3_		510
SufaceCaCO3- GOL-3L1N-K	4A	SufaceCaCO_3_		8447
	4B	SufaceCaCO_3_-GOL		8446
	4C	SufaceCaCO_3_-3L1N		8305
	4D	SufaceCaCO_3_-GOL-3L1N-K		8303

**Table 2:** More representative hydrogen bonds with respect to the percentage of occupancy (comparison of Simulations 4B and 4D).

Simulation 4D: SufaceCaCO_3_-GOL-3L1N-K			Simulation 4D: SufaceCaCO_3_-GOL-3L1N-K		
Donor	Acceptor	Occupancy	Donor	Acceptor	Occupancy
ARG833	CO3445	100.00%	GOL957	CO3513	100.00%
LYS921	CO3435	76.59%	Simulation 4B: SufaceCaCO3-GOL		
LYS929	CO3441	72.02%	GOL804	CO3523	8.27%
LYS921	CO3149	60.72%	GOL804	CO3591	8.22%
LYS907	CO3656	46.88%	GOL804	CO3205	2.07%

## Sample Availability

Samples of the compounds are not available from the authors.
